# Spatial location of neutralizing and non-neutralizing B cell epitopes on domain 1 of ricin toxin’s binding subunit

**DOI:** 10.1371/journal.pone.0180999

**Published:** 2017-07-10

**Authors:** Yinghui Rong, Greta Van Slyke, David J. Vance, Jennifer Westfall, Dylan Ehrbar, Nicholas J. Mantis

**Affiliations:** 1 Division of Infectious Disease, Wadsworth Center, New York State Department of Health, Albany, New York, United States of America; 2 Department of Biomedical Sciences, University at Albany School of Public Health, Albany, New York, United States of America; CEA (Atomic and alternative energies commission), FRANCE

## Abstract

Ricin toxin’s binding subunit (RTB) is a galactose-/N-acetylgalactosamine (Gal/GalNac)-specific lectin that mediates uptake and intracellular trafficking of ricin within mammalian cells. Structurally, RTB consists of two globular domains, each divided into three homologous sub-domains (α, β, γ). In this report, we describe five new murine IgG monoclonal antibodies (mAbs) against RTB: MH3, 8A1, 8B3, LF1, and LC5. The mAbs have similar binding affinities (K_D_) for ricin holotoxin, but displayed a wide range of *in vitro* toxin-neutralizing activities. Competition ELISAs indicate that the two most potent toxin-neutralizing mAbs (MH3, 8A1), as well as one of the moderate toxin-neutralizing mAbs (LF1), recognize distinct epitopes near the low affinity Gal recognition domain in RTB subdomain 1α. Evaluated in a mouse model of systemic ricin challenge, all five mAbs afforded some benefit against intoxication, but only MH3 was protective. However, neither MH3 nor 24B11, another well-characterized mAb against RTB subdomain 1α, could passively protect mice against a mucosal (intranasal) ricin challenge. This is in contrast to SylH3, a previously characterized mAb directed against an epitope near RTB’s high affinity Gal/GalNac recognition element in sub-domain 2γ, which protected animals against systemic and mucosal ricin exposure. SylH3 was significantly more effective than MH3 and 24B11 at blocking ricin attachment to host cell receptors, suggesting that mucosal immunity to ricin is best imparted by antibodies that target RTB’s high affinity Gal/GalNac recognition element in subdomain 2γ, not the low affinity Gal recognition domain in subdomain 1α.

## Introduction

**Fig 1:**
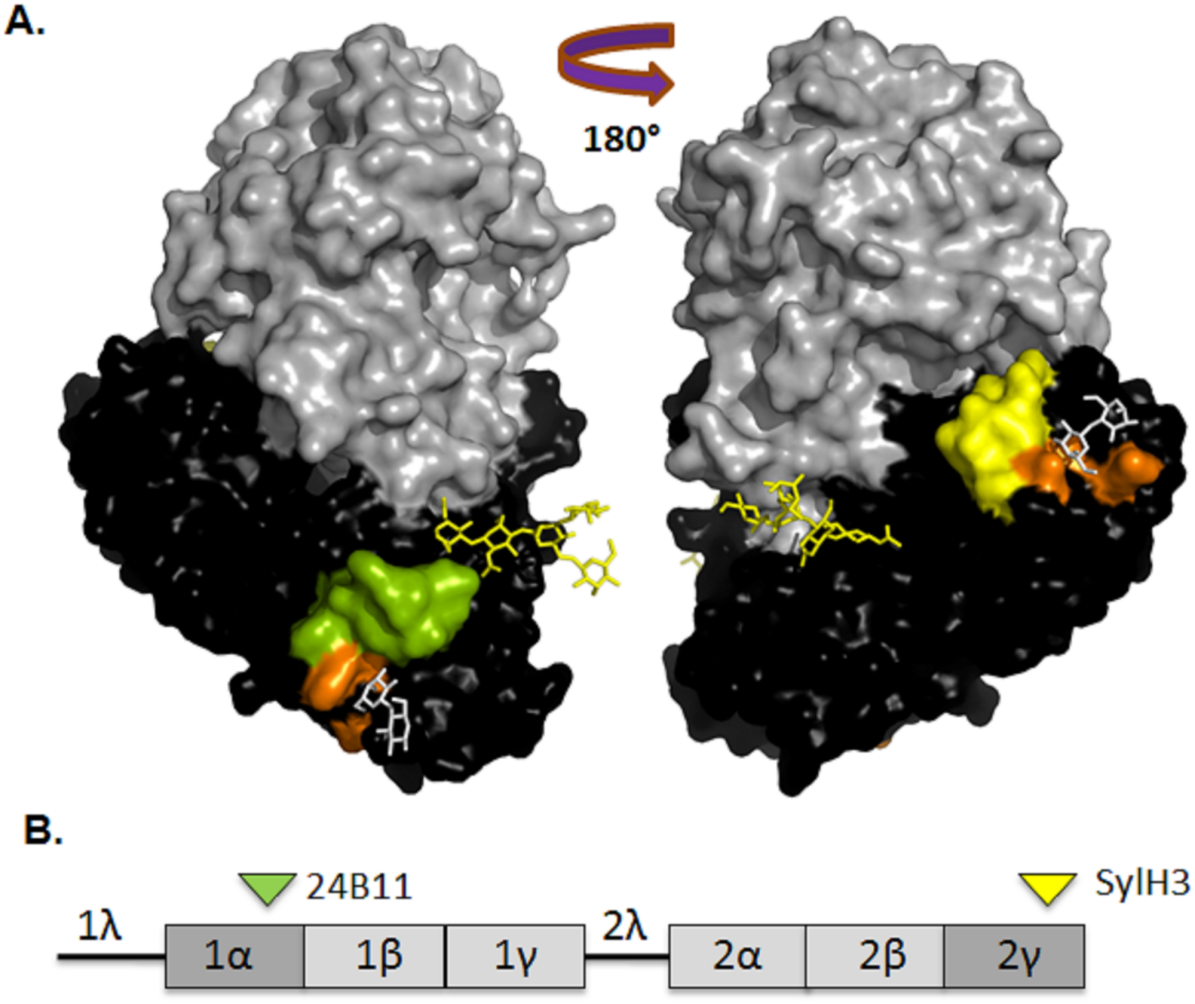
Localization of neutralizing epitopes on RTB. **(A)** A 3D surface representation of ricin toxin derived using PyMOL (PDB ID 2AAI). The following elements are highlighted: RTA (grey), RTB (black), putative 24B11 epitope (green), putative SylH3 epitope (yellow), CRD (orange), N-linked mannose side chains (yellow sticks), and lactose moieties (white sticks). (B) Linear depiction of RTB showing domains (1 and 2), as well as individual sub-domains (1α, 1β, 1γ, 2α, 2β, 2γ). 1λ is a peptide linker connecting RTA to RTB in the ricin pre-protein, while 2λ connects the two RTB domains. The green and yellow arrowhead indicate proposed location of 24B11 and SylH3’s epitope, respectively.

Ricin toxin exposure by injection or inhalation triggers immediate severe local and systemic inflammatory responses that are accompanied by widespread necrosis and fibrosis [1, 2]. Uptake of ricin into mammalian cells, including lung epithelial cells, is mediated by ricin’s binding subunit (RTB), a galactose- and N-acetylgalactosamine (Gal/GalNAc)-specific lectin. RTB is 262 residues in length and, when depicted in linear form, is neatly divided into two domains that are further apportioned into three homologous subdomains (α, β, γ) (Fig 1) [3, 4]. Structurally, RTB has been compared to a dumbbell (70 Å in length) with a low affinity Gal-specific carbohydrate recognition domain (CRD) located in subdomain 1α and a high affinity Gal/GalNAc CRD located in sub-domain 2γ [4–6]. The two CRDs act non-cooperatively and, to some extent, are functionally redundant, as genetic ablation of either one of the CRDs results in a 20–40 fold reduction in ricin cytotoxicity on Vero cells [5, 7]. Ablation of both the CRDs reduced the potency of ricin even further.

We are interested in the development of single or combinations of monoclonal antibodies (mAbs) against ricin’s A and B subunits that could be used therapeutically or prophylactically a means to counter act the effects of ricin, in the event that it were to be used as a bioterrorism agent [8–10]. Several toxin-neutralizing mAbs have been identified that bind (or are proposed to bind) epitopes within RTB subdomains 1α and 2γ [11–16]. The best characterized among these are mAbs 24B11 and SylH3. 24B11 is proposed to recognize a non-linear epitope spanning residues ~35–45 in subdomain 1α [13, 16], while SylH3 is proposed to recognize a discontinuous epitope in close proximity to the CRD located in subdomain 2γ [17]. A second SylH3-like mAb known as JB4 has also been described [16]. *In vivo* studies comparing 24B11 and SylH3 have indicated that the two mAbs (and Fab fragments) can passively protect mice against lethal doses of ricin administered by injection [15]. However, the two mAbs are proposed to function by different mechanisms: SylH3 appears to neutralize ricin by blocking attachment to host cell receptors, whereas 24B11 is proposed to interfere with ricin intracellular transport [17, 18].

Sorting out the relative contributions of RTB’s two domains in ricin pathogenesis and elucidating the mechanisms by which antibodies against RTB interfere with toxin function have important implications for the development of effective ricin therapeutics and mucosal vaccines. Moreover, based on what is known about other toxins like botulinum neurotoxin [19] and staphylococcal enterotoxin [20], for example, it is reasonable to expect that the most effective prophylactics and/or therapeutics for ricin may involve combinations of mAbs. We have recently humanized a potent toxin-neutralizing RTA-specific mAb known as PB10 that protects mice against intranasal and systemic ricin challenge, presumably by interfering with intracellular transport of ricin [21, 22]. Preliminary *in vitro* studies indicate that PB10 can indeed synergize with either 24B11 or SylH3 (B. Mooney and N. Mantis, *manuscript in preparation*), thereby justifying the search for additional RTB-specific mAbs.

Towards this end, we now report the production and characterization of five new RTB-specific murine IgG mAbs: MH3, 8A1, 8B3, LF1, and LC5. The mAbs bound ricin with similar affinities, but displayed a range of *in vitro* and *in vivo* toxin-neutralizing activities, underscoring the importance of epitope specificity as a determinant of antibody function. Competition ELISAs indicate that the two most potent toxin-neutralizing mAbs (MH3, 8A1), as well as one of the moderate toxin-neutralizing mAbs (LF1), recognize distinct epitopes near 24B11’s binding site on RTB subdomain 1α. Like 24B11, passive immunization of mice with MH3 afforded some benefit against a systemic ricin challenge but not against mucosal (intranasal) ricin exposure. This contrasts with SylH3, a previously characterized mAb directed against an epitope near RTB’s high affinity Gal/GalNac recognition element in sub-domain 2γ, which protected animals against systemic and mucosal ricin exposure. SylH3 was significantly more effective than MH3 and 24B11 at blocking ricin attachment to host cell receptors, suggesting that mucosal immunity to ricin is best imparted by antibodies that target RTB’s high affinity Gal/GalNac recognition element in subdomain 2γ, not the low affinity Gal recognition domain in subdomain 1α.

## Materials and methods

### Chemicals, biological reagents and cell lines

Ricin toxin (*Ricinus communis* agglutinin II), *Ricinus communis* agglutinin I (RCA-I), ricin toxin A subunit (RTA), and ricin toxin B subunit (RTB) were purchased from Vector Laboratories (Burlingame, CA). Ricin was dialyzed against phosphate buffered saline (PBS) at 4°C in 10,000 MW cutoff Slide-A-Lyzer dialysis cassettes (Pierce, Rockford, IL) prior to use in *in vitro* and *in vivo* neutralizing assays. GlutaMax^™^, fetal calf serum and goat serum were purchased from Gibco-Invitrogen (Carlsbad, CA). A ClonaCell HY^™^ kit for hybridoma production was purchased from STEMCELL Technologies (Vancouver, BC, Canada). Unless noted otherwise, all other chemicals, including asialofetuin (ASF) were obtained from MilliporeSigma (St. Louis, MO). Vero cells were purchased from the American Type Culture Collection (Manassas, VA). Cell culture media were prepared by the Wadsworth Center Media Services facility. Unless otherwise noted, all cell lines and hybridomas were maintained in a humidified incubator at 37°C with 5% CO_2_.

### Animal care and ethics statement

Experiments described in this study that involve mice were reviewed and approved by the Wadsworth Center’s Institutional Animal Care and Use Committee (IACUC) under protocol #16–384. The Wadsworth Center complies with the Public Health Service Policy on Humane Care and Use of Laboratory Animals and was issued assurance number A3183-01. The Wadsworth Center is fully accredited by the Association for Assessment and Accreditation of Laboratory Animal Care (AAALAC). Obtaining this voluntary accreditation status reflects that Wadsworth Center’s Animal Care and Use Program meets all standards required by law, and goes beyond the standards as it strives to achieve excellence in animal care and use. Mice were euthanized by carbon dioxide asphyxiation followed by cervical dislocation, as recommended by the Office of Laboratory Animal Welfare (OLAW), National Institutes of Health.

### Production and screening of anti-RTB murine mAbs

Groups of female BALB/c mice (Taconic Laboratories, Germantown, NY) were immunized three times with ricin toxoid consisting of RiVax^™^, an attenuated derivative of RTA [23], in association with RTB (D. Vance and N. Mantis, *manuscript in preparation*). The RiVax-RTB immunogen was made by combining equimolar amounts of RiVax (lot 190-100L-GLP-FF-090105, kindly provided by Soligenix, Inc) and RTB (Vector Labs, Burlingame, CA) and incubated on a rotator at 4°C for a week. Covalent association of a fraction of the total RiVax and RTB protein populations was evident by SDS-PAGE. The RiVax-RTB complex (~65 kDa) was concentrated and buffer exchanged using a 50 kilo Dalton molecular weight cut off Amicon Ultra spin filter (Millipore, Billerica, MA). The final concentration of RiVax-RTB complex was determined by sandwich ELISA using ricin as a standard. For immunizations, RiVax-RTB (5 μg) was adsorbed to Alhydrogel aluminum salts (0.85 mg/mL; Brenntag, Mülheim an der Ruhr, Germany) for 2 h at 4°C before being administered to mice by intraperitoneal injections on days 0, 10 and 20. The mice were challenged with ricin on day 58 and their spleens were collected on day 63 for generation of B cell hybridomas, as described [16, 17]. Hybridoma supernatants were screened for reactivity ricin, RTB, and RCA-1 by indirect enzyme linked immunosorbant assays (ELISA) [17]. Murine mAbs were purified by Protein A chromatography at the Dana Farber Cancer Institute (DFCI) Monoclonal Antibody Core facility (Boston, MA).

### ELISA

ELISAs using ricin, RTB, RTB-ASF or RTB pepscan analysis were done as previously described [16, 17]. Nunc Maxisorb F96 microtiter plates (ThermoFisher Scientific, Pittsburgh, PA) were coated overnight with ricin (0.1 μg/well; 15 nM), RCA-I (0.1 μg/well; 8 nM), or RTB (0.1 μg/well; 29 nM), before being treated with hybridoma supernatants or purified mAbs. Horseradish peroxidase (HRP)-labeled goat anti-mouse IgG-specific polyclonal antibodies (SouthernBiotech, Birmingham, AL) were used as the secondary reagent. The ELISA plates were developed using 3,3′,5,5′-tetramethylbenzidine (TMB; Kirkegaard & Perry Labs, Gaithersburg, MD) and analyzed with a SpectroMax 250 spectrophotometer equipped with Softmax Pro 5.4 software (Molecular Devices, Sunnyvale, CA).

To determine if mAbs recognize receptor bound RTB, microtiter plates were coated with asialofetuin (ASF; SigmaMillipore) for 18 h at 4°C, then washed, blocked with 2% goat serum, and overlaid with RTB (10 μg/ml) for 1 hr. The plates were washed again and probed with two fold serial dilutions of mAbs (starting from 5 μg/ml). Plates were then developed using with goat anti-mouse IgG-HRP and TMB, as described above.

We also performed immunocompetition sandwich ELISAs. In this assay, Nunc Immuno MicroWell 96 well plates were coated overnight with a capture anti-RTB mAbs (1 μg/ml). The following day the plates were blocked with 2% goat serum, washed and then overlaid with a mixture of biotin-tagged ricin and competitor mAb (10 μg/ml). The amount of biotin-ricin used in the competition ELISA was adjusted to the EC_90_ for each capture mAb (range: 100–150 ng/ml). After 1 h incubation, plates were washed and developed with streptavidin-HRP antibody (1:500; SouthernBiotech) and TMB, as described above for ELISAs. The percent (%) inhibition of ricin binding to the capture mAb in the present of a competitor mAb was calculated from the optical density (OD) values as follows: 1- value OD_450_ (biotin-ricin + competitor mAb)/ value OD_450_ (biotin-ricin without competitor mAb) x 100.

### Vero cell cytotoxicity assays

Vero cell cytotoxicity assays were performed as previously described [24, 25]. Vero cells were detached from culture dishes with trypsin, then adjusted to ~5 x10^4^ cells per ml, and seeded (100 μl/well) into white 96-well plates (Corning Life Sciences, Corning, NY) and allowed to adhere overnight. The cells were then treated with ricin (0.01 μg/ml; 154 pM), ricin:mAb mixtures, or medium alone (negative control) for 2 h at 37°C. The cells were washed and then incubated for 48 h, at which time cell viability was assessed using CellTiter-GLO (Promega, Madison, WI). All treatments were performed in triplicate, and 100% viability was defined as the average value obtained from wells in which cells were treated with medium only.

### Surface plasmon resonance (SPR)

MAb association and dissociation rates for ricin toxin was determined by surface plasmon resonance (SPR) using the ProteOn XPR36 (Bio-Rad Inc., Hercules, CA), essentially as described [26]. For ricin immobilization, general layer compact (GLC) chips were equilibrated in running buffer (PBS-0.005% Tween) at a flow rate of 30 μl/min. Following EDAC (200 mM) sulfo-NHS (50 mM) activation (3 min), ricin was diluted in 10 mM sodium acetate (pH 5.0) at either 4 μg/ml or 2 μg/ml and allowed to be immobilized for 2 min. A third vertical channel received only acetate buffer and served as a reference channel. The surfaces were deactivated using 1M ethanolamine for 5 min. The ProteOn MCM was rotated to the horizontal orientation for affinity determination experiments. Each mAb was serially diluted in running buffer and then injected at 50 μl/min for 180s, followed by 1 to 3 hr of dissociation. After each experiment, the chip regenerated with 10 mM glycine [pH 1.5] at 100 μl/min for 18 s, until the RU values had returned to baseline. All kinetic experiments were performed at 25°C. Kinetic constants for the antibody/ricin interactions were fitted using the Langmuir 1:1 binding model available in the ProteOn Manager software 3.1.0 (Bio-Rad Inc.) [27].

### Passive protection studies

Animal studies were conducted under strict compliance with the Wadsworth Center’s Institutional Animal Care and Use Committee (IACUC), as indicated above. RTB mAbs (5 μg or 25 μg) were diluted in endotoxin-free PBS and then administered in a final volume of 0.4 ml to female BALB/c mice (ages 8–10 weeks) by i.p. injection. Twenty-four h later, the mice received the equivalent of 10xLD_50_ for ricin (~2 μg per mouse) delivered by i.p. or intranasal (i.n.) routes. Following ricin challenge, animals were monitored twice daily for 7 days for symptoms of ricin intoxication, including hypoglycemia [28]. Mice were euthanized when they became overtly moribund and/or blood glucose levels fell below 25 mg/dl, as mandated by the Wadsworth Center’s IACUC.

### Statistical analyses and molecular modeling

Statistical analysis was carried out using GraphPad Prism 5 (GraphPad Software, San Diego, CA) or R version 3.3.1[29]. Fisher’s exact test was used to compare the proportions of surviving mice in each group to the control group. The test was corrected for multiple comparisons via the Bonferroni method; a *p*-value of 0.007 was used as a cut-off to judge significance. Cox regression analysis was used to calculate hazard ratios and their corresponding 95% confidence intervals for each group [30, 31]. PyMOL Molecular Graphics System (Version 1.8 Schrödinger, LLC) was used for modeling and annotating epitopes on ricin toxin (PDB ID 2AAI) [32].

## Results and discussion

### Isolation and preliminary characterization of RTB-specific mAbs

**Fig 2:**
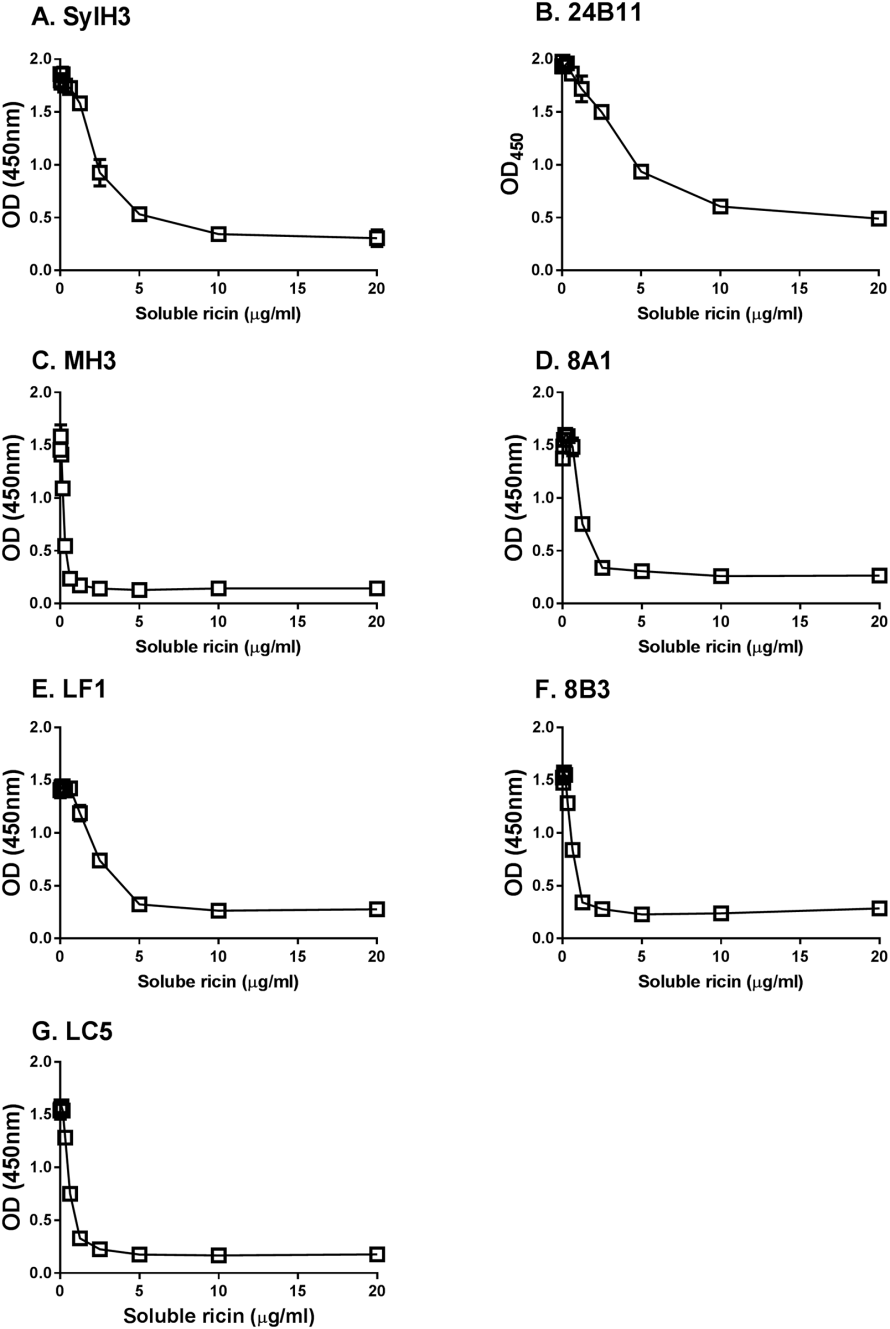
Recognition of soluble ricin by RTB-specific mAbs. Individual RTB-specific mAbs **(panels A-G)** were mixed with serial dilutions of soluble ricin (as indicated on the x-ordinate), then applied to ricin-coated microtiter plates. The plates were developed with an HRP-labeled goat anti-mouse IgG-specific secondary antibody and TMB substrate to detect amount of mAb bound in each well (y-ordinate). The results shown are a single representative experiment in which each sample was done in duplicate. Error bars, when visible, reflect the variation between technical replicates.

**Fig 3:**
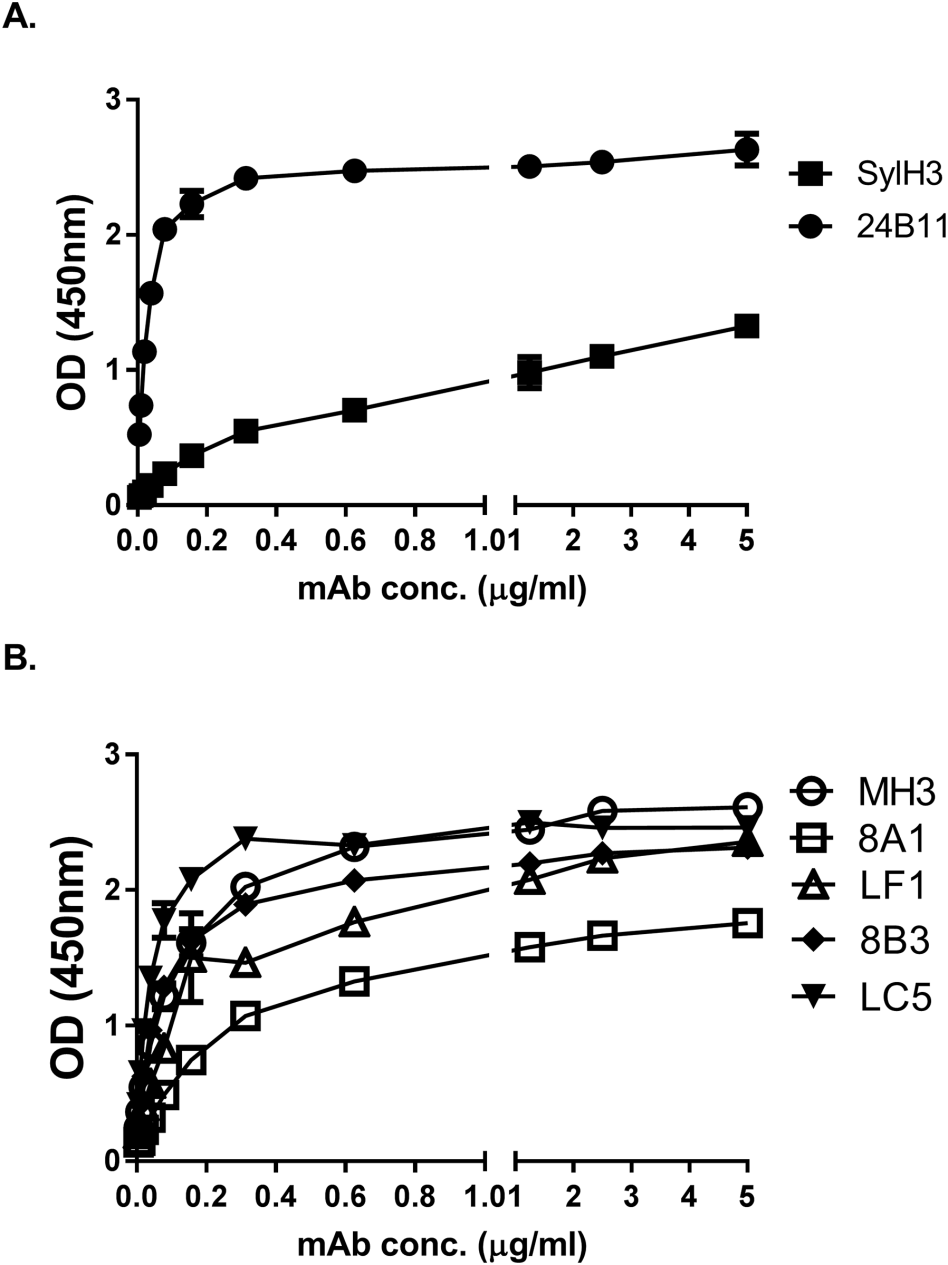
Recognition of receptor bound RTB by MH3, 8A1, 8B3, LF1, and LC5. Ninety-six well microtiter plates were coated with ASF-RTB and then probed with indicated mAbs (starting at 5μg/ml): (top panel) **SylH3** and **24B11**; (bottom panel) **MH3**, **8A1**, LF1, 8B3, and LC5. The results shown are a single representative experiment in which each sample was done in duplicate. Error bars, when visible, reflect the variation between technical replicates.

In an effort to isolate additional RTB-specific mAbs against RTB, groups of BALB/c mice were immunized as described in the Materials and Methods. Spleens from mice with the highest ricin-specific endpoint titers were collected for generation of B cell hybridomas and screened for reactivity with ricin, RTB, and RCA-1 (see sections below). The screen yielded five IgG_1_ mAbs of interest: MH3, 8A1, 8B3, LF1, and LC5 (Table 1). Each of the five mAbs recognized ricin by direct ELISA (S1 Fig), as well as ricin in solution (Table 1; Fig 2). They were all specific for RTB, as demonstrated by an RTB-ASF ELISA in which RTB was captured onto the microtiter plates via a surrogate receptor, ASF (Fig 3). ASF is routinely used as a receptor for RTB because it displays three Asn-linked triantennary complex carbohydrate chains with terminal Gal and GalNac moieties [7, 33, 34]. All five of the mAbs likely recognize discontinuous (conformation-dependent) epitopes, as they were negative for reactivity in an RTB peptide array (pepscan) (S2 Fig; *data not shown*).

**Table 1:** Properties of new anti-RTB mAbs compared to 24B11 and SylH3.

mAb	EC_50_ ^c^	Relative Kinetic Parameters ^a^			IC_50_ ^d^	Competition ^b^	
		k_a_ (1/Ms)	k_d_ (1/s)	K_D_ (M)		SylH3	24B11
SylH3	2.5	2.20 x 10^5^	8.06 x 10^−6^	3.67 x10^-11^	12	+	-
24B11	5	2.67 x 10^5^	1.51 x 10^−5^	5.64 x 10^−11^	1.2	-	+
**MH3**	0.3	2.58 x 10^5^	7.45 x 10^−6^	2.89 x10^-11^	1.6	-	+
**8A1**	2.5	3.67 x 10^5^	2.75 x 10^−5^	7.49 x10^-11^	3.3	-	+
8B3	2.5	4.78 x 10^5^	4.25 x 10^−5^	8.89 x10^-11^	27	+	+
LF1	2.5	1.02 x 10^5^	6.92 x 10^−6^	6.79 x10^-11^	27	-	+
LC5	0.6	4.96 x 10^5^	8.95 x 10^−5^	1.8 x10^-10^	-	-	-

^*a*^, apparent binding association (k_a_) and dissociation (k_d_) rate constants, as well as apparent equilibrium dissociation constants (K_D_), were determined by SPR using IgG molecules (not Fab fragments) and ricin toxin-coated GLC chips. The Langmuir model was used to analyze sensorgram results, as described in the Materials and Methods;

^*b*^, competition ELISAs are described in the Materials and Methods. The minus (-) symbol indicates that binding inhibition values were <10% of controls;

^*c*^ As determined in the soluble ricin competition assay, described in Fig 3;

^*d*^, nM.

The relative avidities of each of the five RTB-specific IgG mAbs for ricin holotoxin was determined by SPR and compared to SylH3 and 24B11. Four of the five mAbs had apparent dissociation constants (K_D_) that were similar to SylH3 and 24B11. LC5’s had the weakest apparent K_D_ as compared to the other mAbs, due to a faster off rate (k_d_) (Table 1; S3 Fig).

To examine toxin-neutralizing activity (TNA), MH3, 8A1, 8B3, LF1, and LC5 were tested in a Vero cell-based cytotoxicity assay alongside SylH3 and 24B11. The five new mAbs stratified into three groups: two with strong TNA (MH3, 8A1), two with moderate TNA (LF1, 8A3), and LC5, which lacked any detectable TNA (Table 1; S4 Fig). MH3 and 8A1 had IC_50_s that were similar to those of SylH3 and 24B11, while LF1 and 8A3 had IC_50_s that were 6–12 times higher than SylH3 and 24B11. For simplicity sake, the strong neutralizers will be depicted in **bold** and the moderate toxin-neutralizing mAbs will be underlined for the remainder of this article.

### Epitope localization of RTB-specific mAbs

To tentatively localize the epitopes recognized by the five new mAbs to either RTB subdomain 1α or subdomain 2γ, we performed competition ELISAs with 24B11 and SylH3. The competition assay tested the ability of plate bound 24B11 or SylH3 to “capture” soluble biotin-labeled ricin in the absence or presence of competitor mAbs. A reduction in the amount of ricin captured by 24B11 or SylH3 in the presence of competitor mAbs was interpreted as being the result of direct epitope overlap or steric inhibition [35].

Three of the mAbs, **MH3**, **8A1** and LF1, competitively inhibited biotin-labeled ricin from being captured by plate bound 24B11, but did not affect SylH3’s ability to capture ricin in the same assay (Table 1), suggesting that the two strong (**MH3**, **8A1**) and one moderate (LF1) toxin-neutralizing antibodies recognize epitopes on RTB subdomain 1α. Localization of the 8B3’s epitope was confounded by the fact that it competed with both 24B11 and SylH3 (Table 1). Conversely, LC5 did not interfere with either 24B11 or SylH3, suggesting it recognizes an epitope within one of RTB internal subdomains (Table 1; Fig 1).

To refine the spatial location of the epitopes on RTB recognized by **MH3**, **8A1**, 8B3, LF1, and LC5, we conducted cross-competition sandwich ELISAs in which each mAb was tested as both capture (plate bound) and competitor (solution). As shown in Table 2, each of the mAbs was effective (79–97%) at blocking themselves in the ELISA, thereby validating the competition assay. Moreover, 24B11 and SylH3 did not compete with each other, an outcome consistent with the two mAbs recognizing epitopes on opposite poles of RTB.

**Table 2:** Epitope localization by mAb competitive binding assays.

**mAb** ^a^	SylH3	24B11	**MH3**	**8A1**	8B3	LF1	LC5
SylH3	97	<10	<10	<10	85	<10	<10
24B11	<10	96	91	55	92	95	<10
**MH3**	<10	37	89	<10	43	34	<10
**8A1**	<10	35	<10	81	45	57	<10
8B3	87	79	86	21	92	93	<10
LF1	<10	50	56	26	62	95	<10
LC5	<10	<10	<10	<10	<10	<10	79

^*a*^, competitive sandwich ELISAs were conducted as described in the Materials and Methods.

The capture mAbs (1 μg/ml) listed in the top row were coated on the 96 well microtiter plates, while the competitor mAbs (10 μg/ml) listed in the far left column were mixed with biotin-tagged ricin (equivalent to the EC_90_ for each coated mAb) in solution then applied to the microtiter plates. The numbers are the % inhibition of ricin capture, as defined in the Materials and Methods.

We now discuss the proposed spatial localization of each of the new RTB-specific mAbs in turn, based on the cross-competition ELISA results in Table 2:

**MH3**: When evaluated as a capture mAb (plate bound), **MH3** was strongly impacted by 24B11 (91%) and 8B3 (86%), partially by LF1 (56%), and not affected by **8A1** or LC5. Thus, MH3’s epitope is most likely positioned in close proximity to 24B11 and 8B3 binding sites.

**8A1**: When evaluated as a capture mAb (plate bound), **8A1** was partially inhibited by 24B11 (55%) and only weakly by 8B3 and LF1, suggesting **8A1**’s epitope is peripheral to 24B11’s binding site and partially overlapping with 8A1 and LF1.

8B3: When evaluated as a capture mAb (plate bound), 8B3 was strongly inhibited by 24B11 (92%), and partially by **MH3**, **8A1**, and LF1, suggesting its epitope is in close proximity to 24B11’s epitope and equidistant from the binding sites of the **MH3**, **8A1**, and LF1. However, the ability of 8B3 to capture soluble ricin was also impacted by SylH3, suggesting 8B3 may have a second binding site on the opposite pole of RTB. This possibility will be discussed below.

LF1: When evaluated as a capture mAb (plate bound), LF1 was strongly inhibited by 24B11 (95%) and 8B3 (93%) and partially inhibited by **MH3** and **8A1**. Thus, LF1’s epitope is likely in close proximity to both 24B11 and 8B3 binding sites.

LC5: When evaluated as a capture mAb (plate bound), LC5 did not compete with 24B11 or the other four mAbs described in this study (ie., **MH3**, **8A1**, 8B3, and LF1). Moreover, competition assays between LC5 and additional RTB [16, 17] and RTA-specific mAbs in our collection [36] also proved uninformative (S1 Table). Based on these results we speculate that LC5 recognizes an epitope within one of RTB’s internal domains (i.e., 1β-1γ or 2α-2β).

It should be noted that in the cross-competition ELISAs (Table 2) there are discrepancies in the degree of competition depending on whether the query mAb is used for capture (top row) or competitor (left column). This is most relevant in the case of 24B11: when 24B11 is tested as the capture mAb it is only marginally affected by competitors (**MH3**, **8A1**, 8B3 and LF1) other than itself. In contrast, when 24B11 was used as a competitor (in solution) it strongly interfered (range 55–95%) with the ability of same panel of mAbs (**MH3**, **8A1**, 8B3 and LF1) to capture ricin. We postulate that 24B11 has a larger footprint (epitope) on RTB than the other mAbs (**MH3**, **8A1**, 8B3 and LF1). Thus, when 24B11 is pre-bound to ricin in solution (as a competitor) it effects the capture of ricin by other mAbs. Conversely, with a larger footprint, 24B11 may retain the ability to capture ricin in solution even when RTB is co-occupied by one of the other RTB-specific mAbs like **MH3**, **8A1**, 8B3 and LF1.

**Fig 4:**
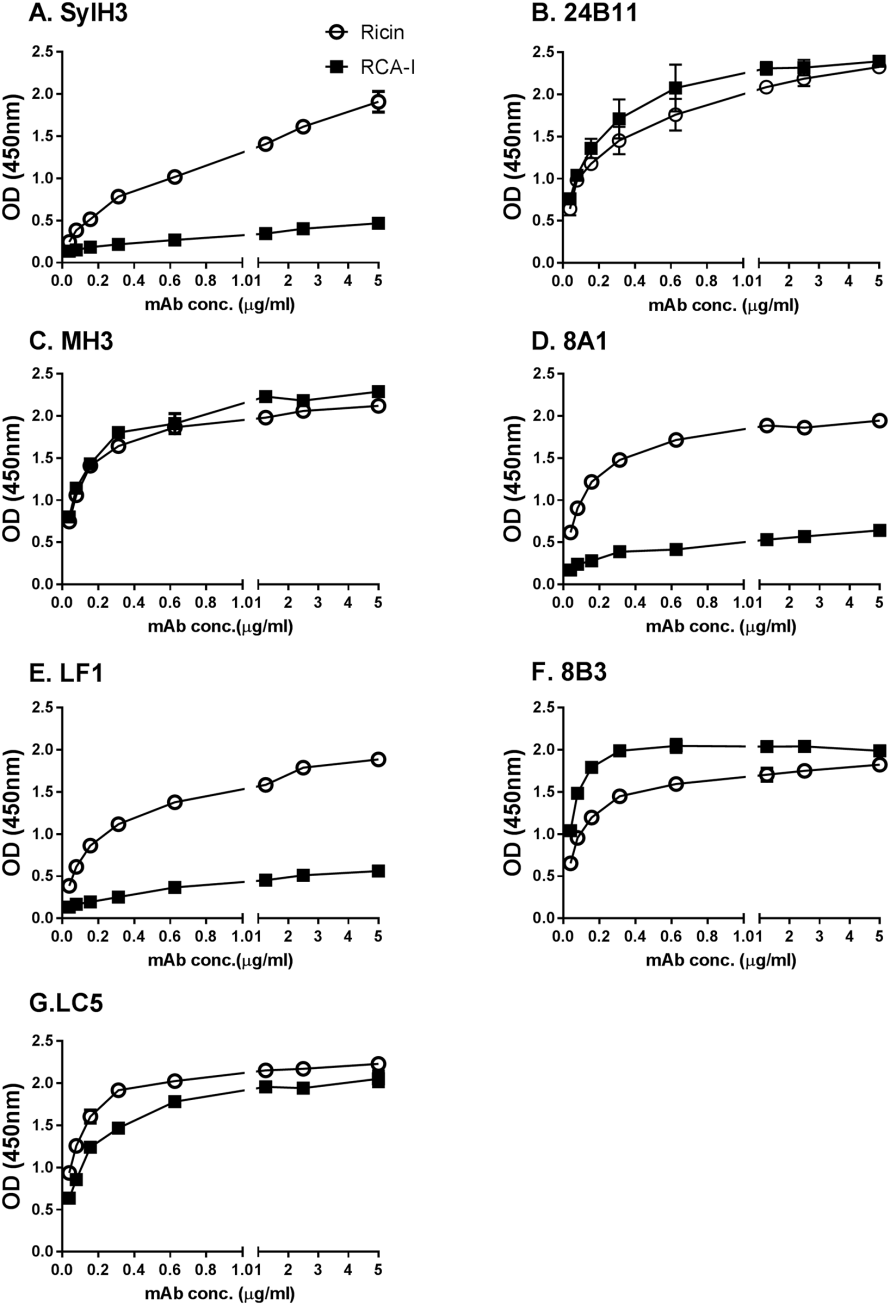
Differential reactivity anti-RTB mAbs with ricin and RCA-I. **(Panels A-F)** Ninety-six well microtiter plates were coated with 1 μg/ml ricin (open symbols) or RCA-I (closed symbols) and probed with indicated mAbs at the concentrations shown on the x-axis. The data (mean ± SD) shown represent a single experiment in which each sample was done in replicate and repeated at least three times. The SD may be too small to visualize in the figure.

As an additional means to localize the epitopes recognized by the five new mAbs, we performed direct ELISAs with mictrotiter plates coated with *Ricinus communis* agglutinin I (RCA-I). RCA-I’s binding subunit, RCB, shares 84% amino acid sequence identity with RTB. Differential binding reactivity of mAbs to RTB and RCB can be used as a tool to pinpoint possible epitopes [16]. By direct ELISA, 24B11 recognizes RTB and RCB equally well [13], whereas SylH3 binds preferentially to RTB (Fig 4) [16]. In the case of the five new mAbs, **MH3**, 8B3, and LC5 bound RTB and RCB equally well, indicating that their epitopes are conserved between ricin toxin and ricin agglutinin. **8A1** and LF1, on the other hand, had little to no reactivity with RCB, indicating that their epitopes are specific to ricin toxin. Based these results, alongside the results of the cross-competition ELISA, we tentatively localized the epitopes recognized by **MH3**, **8A1**, 8B3 and LF1 onto the surface of RTB (S5 Fig).

### Passive protection studies with RTB-specific mAbs

We next examined the capacity of **MH3**, **8A1**, 8B3, LF1, and LC5, to passively protect mice against systemic ricin toxin challenge. Ricin (2 μg; 10 x LD_50_) was mixed with 5 or 25 μg of each mAb and then administered to mice by intraperitoneal injection [16]. 24B11 and SylH3 were used as positive controls. Mice were monitored for up to seven days following toxin challenge. While all five mAbs extended the experimental animal’s mean time to death at high dose, as compared to control ricin-only mice, only **MH3** conferred a statistical degree of protection when survival was the endpoint (Table 3; S6 Fig). To determine whether MH3 can also protect against mucosal toxin exposure, ricin (2 μg) was mixed with 400 μg MH3, 24B11, SylH3 or LC5 and then administered to mice by the intranasal route. We have shown previously that 10–20 times higher serum antibody levels are required to protect mice against intranasal as compared to intraperitoneal challenge [21]. In the intranasal challenge model, we found that SylH3 protected 80% of the mice, whereas 24B11 and **MH3** only protected 20% of the animals (Table 3; S6 Fig). As expected, LC5 afforded no protection against mucosal ricin challenge.

**Table 3:** mAb passive protection studies in mouse models of systemic and mucosal ricin challenge.

Intraperitoneal				Intranasal		
Group	Survivors	*p*-value^a^	HR (95% CI)^b^	Survivors	*p*-value^a^	HR (95% CI) ^b^
SylH3	9/10	0.0001	0.0022 (0.0002–0.0190)	8/10	0.0007	0.0280 (0.0054–0.1438)
24B11	3/5	0.0275	0.0096 (0.0018–0.0510)	1/5	0.3333	0.1457 (0.0415–0.5112)
MH3	7/10	0.0031	0.0098 (0.0029–0.0335)	2/10	0.4737	0.2227 (0.0791–0.6270)
8A1	1/5	0.3571	0.0577 (0.0157–0.2120)	-	-	-
8B3	3/10	0.2105	0.0289 (0.0100–0.0836)	-	-	-
LF1	0/10	>0.9999	0.1171 (0.0465–0.2951)	-	-	-
LC5	0/5	>0.9999	0.1848 (0.0707–0.4828)	0/5	>0.9999	0.8302 (0.2803–2.4585)*
Control	0/9	-	-	0/10	-	-

^*a*^, *p*-values of Fisher’s exact tests comparing survival between individual experimental groups and the control group.

^*b*^, in all cases the Cox regression analysis indicated that mAb treatment was significantly beneficial (*p*-values <0.01) except for LC5 treatment in the intranasal group (*p* = 0.74).

**Fig 5:**
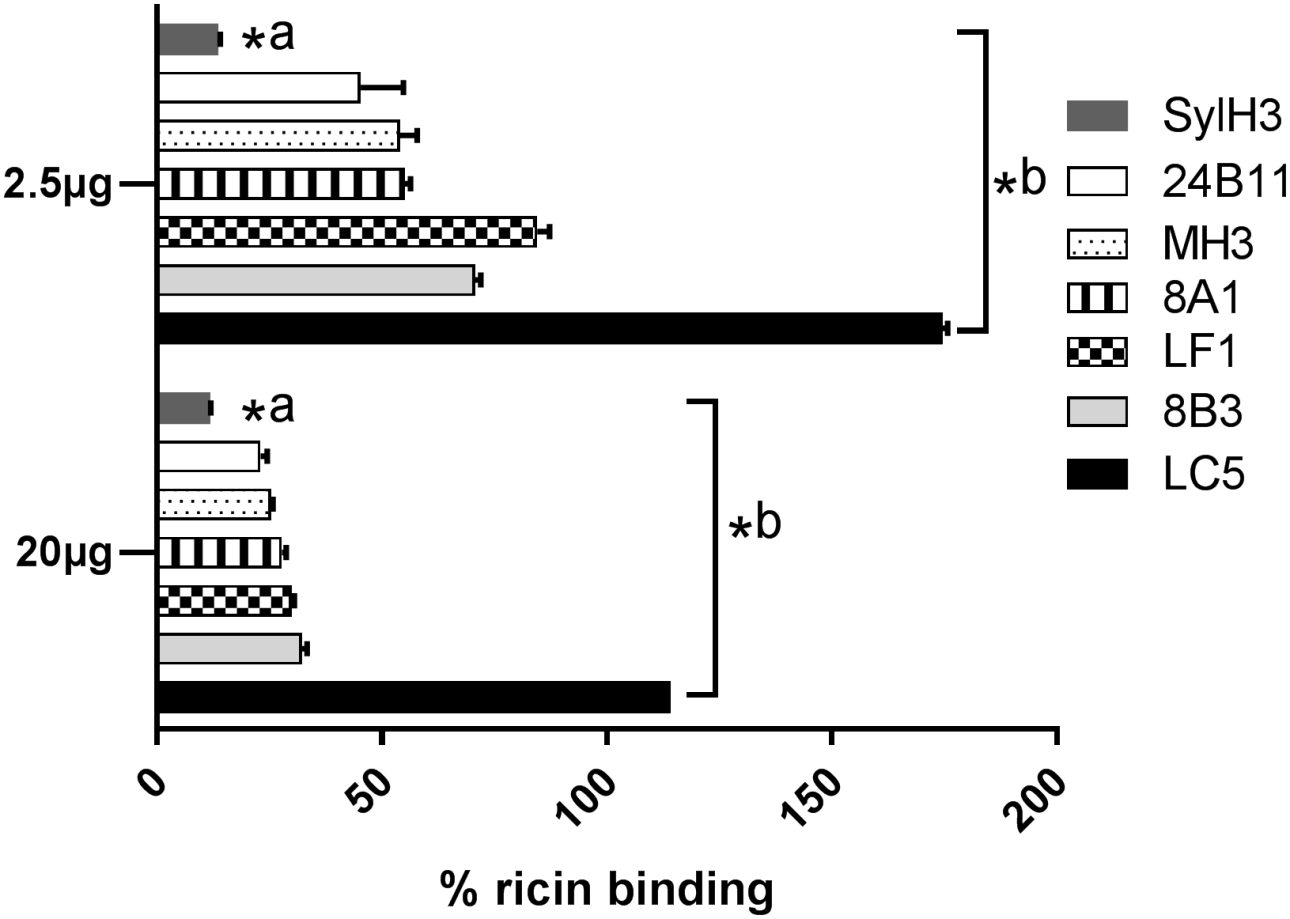
Interference with ricin-receptor interactions by anti-RTB mAbs. Biotin-labeled ricin (50 ng/ml) was mixed with indicated mAbs (2.5 μg or 20 μg) and then applied to 96-well micotiter plates coated with ASF (4μg/ml), as described in Material and Methods. Biotin-ricin binding to ASF was detected with avidin-HRP and TMB substrate. Biotin-ricin binding was then normalized to biotin-labeled ricin in the absence of antibody. The enhanced (>100%) ricin-capture observed in Panel A (at the equivalent of a 2:1 mAb:toxin molar ratio) but not in panel B (at 16:1 mAb:toxin molar ratio) is likely due to a bridging effect in which one arm of LC5 associates with ricin bound to ASF, while the other arm of LC5 captures ricin in solution. At higher LC5 stoichiometric ratios when antibody is in excess such bridging does not occur because soluble ricin is saturated with unbound LC5. The data represent a single experiment in which each sample was done in triplicate (mean ± SD) and repeated at least three times. The asterisks indicate two levels of statistical analysis performed on this assay: *, ^a^ indicates that the inhibitory effect of SylH3 on ricin binding was significantly greater (*p* < 0.05) that the other mAbs tested; *,^b^ indicates that the inhibitory effect of SylH3, 24B11, MH3, 8A1, LF1 and 8B3 were significantly greater (*p* < 0.05) than LC5.

The passive protection studies are consistent with antibodies against RTB subdomain 1α being less effective than antibodies against subdomain 2γ at neutralizing ricin in the mucosal compartment, which may have to do with the fact that RTB’s high affinity Gal/GalNAc-specific binding element is located in subdomain 2γ and the low affinity Gal-specific element is in 1α [5]. We questioned whether this difference could be explained by antibodies against 1α being less effective than antibodies against subdomain 2γ at interfering with RTB-receptor interactions. Using an ASF binding assay [16], it was indeed apparent that SylH3 was more effective (~90%) than 24B11 or any of newly characterized strong or moderate toxin-neutralizing mAbs (**MH3**, **8A1**, 8B3, and LF1) at inhibiting ricin-ASF engagement (Fig 5). It is tempting to speculate that SylH3 (unlike 24B11 and MH3) protects the lung mucosa from the toxin-induced inflammation and cell death by virtue of its ability to inhibit the earliest events in ricin’s cytotoxic pathway (i.e., receptor attachment).

### Conclusions

In summary, we have produced and characterized five new anti-RTB mAbs: two with strong *in vitro* TNA (**MH3**, **8A1**), two with moderate TNA (8B3, LF1) and one devoid of TNA (LC5). Competitive ELISAs with 24B11 suggest that **MH3**, **8A1** and LFI recognize epitopes within RTB subdomain 1α, close to ricin’s low affinity Gal-specific carbohydrate recognition domain [4, 33]. Unfortunately, the exact location of 24B11’s epitope is tentative at best, as it is based solely on mapping studies done using a phage-display peptide library [13]. Thus, epitope positioning for **MH3**, **8A1**, 8B3, and LF1, as shown in S5 Fig, are not absolute, but rather relative to 24B11. Nonetheless, the competition ELISAs indicate that there are at least four distinct epitopes clustered near the 24B11 binding site on RTB. LC5, by contrast, was not inhibited by any other RTB-specific mAbs in our current library (S1 Table), leaving us unable to ascribe a putative epitope for this specific non-neutralizing antibody.

The position of 8B3’s epitope was confounded by the observation that 8B3 competitively inhibited both SylH3 and 24B11 from binding to ricin. How is it possible that 8B3 interferes with mAbs that bind opposite poles of RTB? We postulate that it may have more than one binding site on RTB. Rutenber and colleagues have demonstrated that RTB’s six subdomains most certainly arose through gene duplication, as evidenced primarily by amino acid similarity across subdomains [4]. There is also precedent for dual antibody binding sites on RTB. The mAb JB11 is proposed to recognize a primary epitope in subdomain 2β (DSNIR) and a degenerate motif in RTB subdomain 1α (DNTIR) [16]. As it pertains to 8B3, there are 12 residues that are conserved between RTB’s subdomains 1α and 2γ, seven of which are surface exposed and therefore putative antibody contact points (S7 Fig). Furthermore, 5 of these are conserved in subdomains 1α and 2γ of RCA-I’s B subunit, possibly explaining 8B3’s ability to recognize RCA-1 (Fig 4F).

Finally, the results of this study confirm that SylH3 is one of the few anti-RTB mAbs able to neutralize ricin in mouse systemic and mucosal challenge models [17]. Indeed, while SylH3’s epitope is not known in detail, it apparently recognizes a neutralizing “hotspot” on ricin situated at the RTA-RTB interface that we now refer to as super Cluster II (A. Poon, D. Vance, and N. Mantis, *unpublished results*). While we have speculated that SylH3 primarily functions by blocking attachment of ricin to host cells, we cannot exclude the possibility it may also interfere (as an immune complex) with intracellular trafficking of ricin [18]. Defining how exactly anti-RTB antibodies neutralizing ricin *in vitro* and *in vivo* has important implications when considering how to best design an immune-based antidote to ricin toxin. Of particular interest are antibodies capable to protecting against aerosolized ricin exposure, which is known to elicit rapid onset of inflammation including hemorrhages, inflammatory exudates, and tissue edema[2].

## Supporting information

S1 Table LC5 competition ELISA. (PDF)

S1 Fig Recognition of ricin toxin byanti-RTB mAbs. Ninety-six well microtiter plates were coated with ricin and then probed with indicated mAbs (starting at 5μg/ml). The results shown are a single representative experiment in which each sample was done in duplicate. Error bars, when visible, reflect the variation between technical replicates.
(PDF)

S2 Fig Representative RTB-pepscan analysis of MH3. MH3 was examined by ELISA for the ability to bind to an RTB peptide array consisting of 32 15-mers (A1-C8, *x*-axis), each overlapping its neighbors by 7 amino acids. MH3 reactivity with RTB is shown in far-right column. The peptide array was performed at least two independent times with similar results. The results shown are from one representative experiment. The OD_450_nm (*y*-axis) values refer to the reactivity of MH3 with specific peptides and were obtained using peptide array ELISA.
(PDF)

S3 Fig Kinetics of ricin toxin binding by anti-RTB mAbs. **(Panels A-G** Sensorgrams from SPR analysis in which ricin-coated chips (4 μg/ml) were probed with indicated RTB-mAbs. The real time binding was recorded as response units (RU) versus time. Binding was determined over a range of RTB mAb concentrations (nM); 60 (red), 20 (blue), 6.6 (green), 2.2 (purple), and 0.7 (yellow). The curves were fit using the Langmuir binding model with the ProteOn Manager software 3.1.0. (BioRad, Inc.).
(PDF)

S4 Fig Representative ricin cytotoxicity assays in the present of anti-RTB mAbs. Ricin was mixed with 2-fold serial dilutions of indicated mAbs (A) SylH3 and24B11; (B) MH3, 8A1, LF1, 8B3, andLC5 and then applied to Vero cells for 2 h. The cells were then washed and cell viability was measured 48 h later, as described in Materials and Methods. The results (mean ± SD) represent a single experiment done in triplicate and repeated at least three times. As needed, the cytotoxicity assays were repeated (data not shown) to generate IC_50_ values.
(PDF)

S5 Fig Proposed epitope locations for 24B11, MH3, 8B3, 8A1, and LF1 on RTB. A surface representation of ricin derived using PyMol (PDB 2AAI) highlighting 24B11’s previously proposed epitope (green) and the relative spatial distribution (red circles) of epitopes recognized by MH3, 8B3, 8A1, and LF1 based on competition ELISAs presented in Table 2. Residues are non-conserved residues between RTB and RCB are colored orange.
(PDF)

S6 Fig Passive protection studies with anti-RTB mAbs. Passive protection studies in which groups of adult BALB/c mice (n = 5 per experiment) were injected intraperitoneally with indicated mAbs and then challenged 24 h later with ricin (2 μg; 10 x LD_50_) by the (**A**) intraperitoneal or (**B**) intranasal route. Survival was monitored for seven days.
(PDF)

S7 Fig Putative duplication of 8B3’s epitope on RTB. (**A**) Manual alignment of subdomains 1α and 2γ from RTB (PDB ID 2AAI) and RCA-I’s B subunit (RCB; PDB ID 1RZO). Boxed in bold, conserved residues; boxed, conservative substitutions; *, surface exposed residues that are conserved/conservative between RTB and RCB. These same residues are colored red in Panels B and C. **(B,C**) PyMol image of ricin (PDB ID 2AAI) showing potential binding sites of 8B3 on subdomains 1α and 2γ of RTB. Residues in red are surface exposed and conserved/conservative between RTB and RCB, as indicted in Panel A.
(PDF)
