# Supplementary material for: Spatial location of neutralizing and non-neutralizing B cell epitopes on domain 1 of ricin toxin’s binding subunit
Source: PLoS One. 2017 Jul 10;12(7):e0180999. doi: 10.1371/journal.pone.0180999 (PMC5507285; doi:10.1371/journal.pone.0180999)
Supplement: S1 Table — (PDF) [file pone.0180999.s001.pdf]

S1 Table. LC5 Competition ELISA

mAb	Target	% Inhibition[a]
LC5	RTB	82
SylH3	RTB	15
24B11	RTB	<10
BJF9	RTB	<10
JB11	RTB	<10
JB4	RTB	<10
GD12	RTA (4)	16
JD4	RTA (4)	<10
WECH1	RTA (2)	<10
SyH7	RTA (2)	<10
PA1	RTA (2)	<10
IB2	RTA (3)	<10

[a], % inhibition of LC5 capture of biotinylated ricin by various mAbs, as described in Materials and Methods.
