# Supplementary figures and images for: Spatial location of neutralizing and non-neutralizing B cell epitopes on domain 1 of ricin toxin’s binding subunit
Source: PLoS One. 2017 Jul 10;12(7):e0180999. doi: 10.1371/journal.pone.0180999 (PMC5507285; doi:10.1371/journal.pone.0180999)

S1 Figure

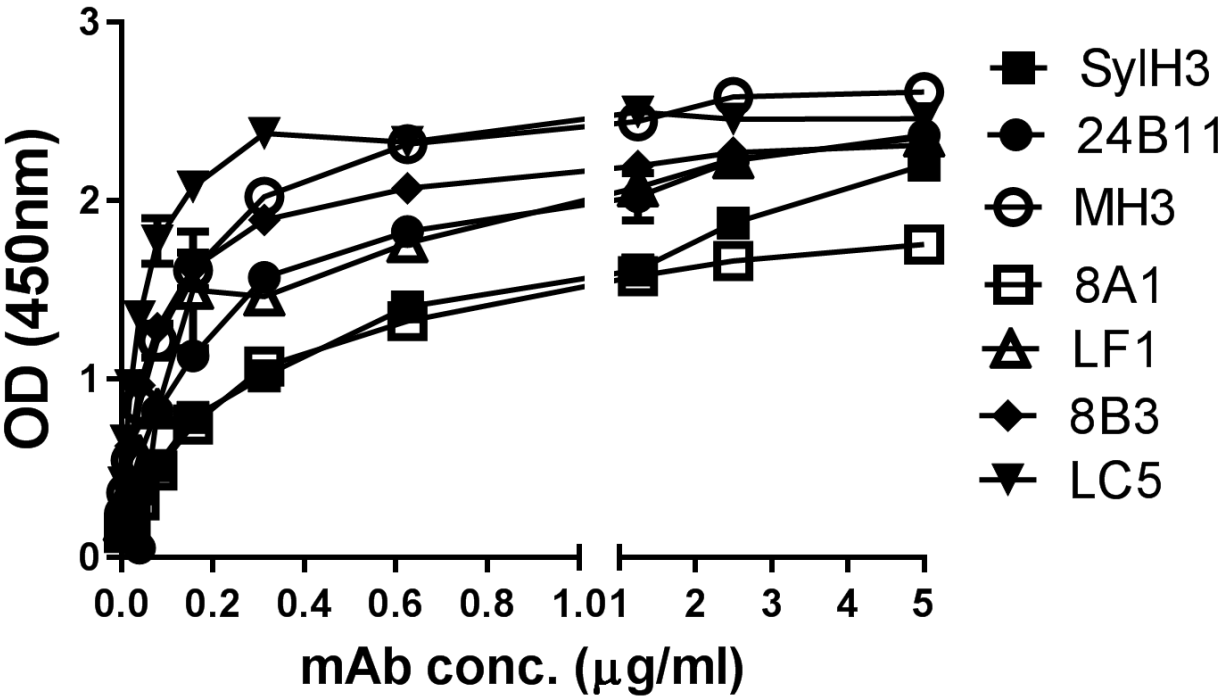

Supplement: S1 Fig — Ninety-six well microtiter plates were coated with ricin and then probed with indicated mAbs (starting at 5μg/ml). The results shown are a single representative experiment in which each sample was done in duplicate. Error bars, when visible, reflect the variation between technical replicates. (PDF) [file pone.0180999.s002.pdf]

S2 Figure

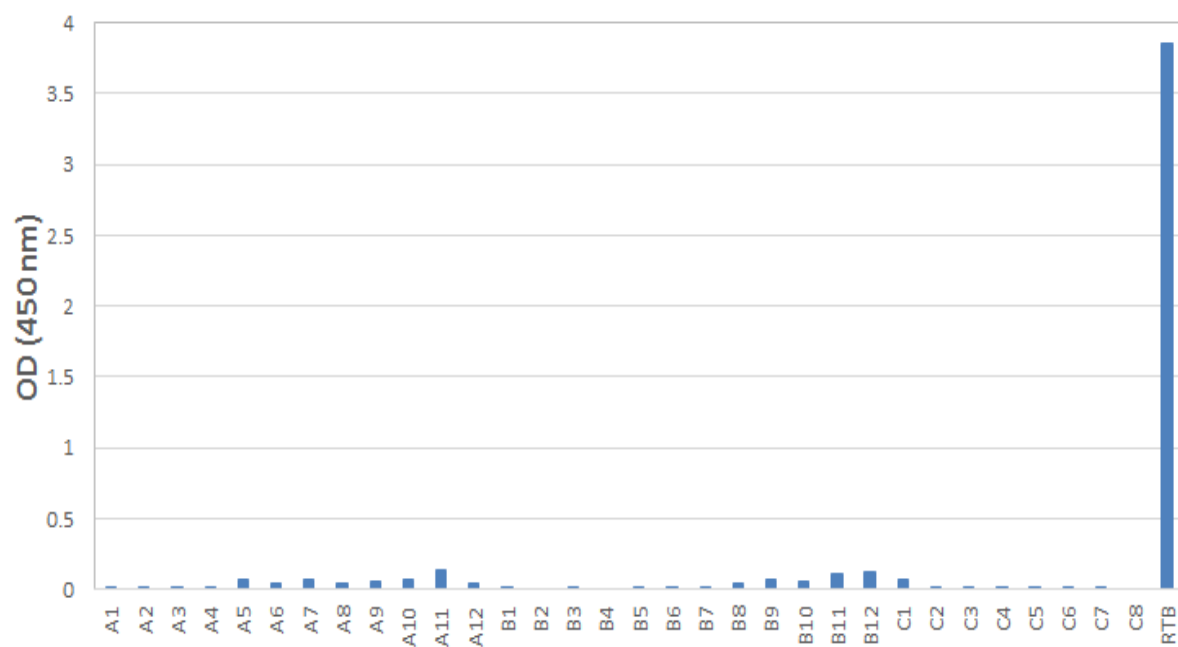

Supplement: S2 Fig — MH3 was examined by ELISA for the ability to bind to an RTB peptide array consisting of 32 15-mers (A1-C8, x-axis), each overlapping its neighbors by 7 amino acids. MH3 reactivity with RTB is shown in far-right column. The peptide array was performed at least two independent times with similar results. The results shown are from one representative experiment. The OD450nm (y-axis) values refer to the reactivity of MH3 with specific peptides and were obtained using peptide array ELISA. (PDF) [file pone.0180999.s003.pdf]

# S3 Figure

## A. SyIH3

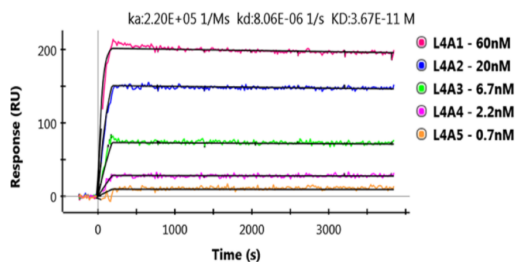

## B. 24B11

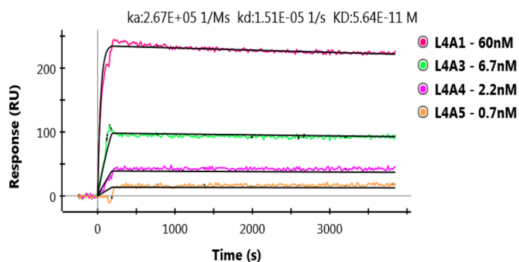

## C. MH3

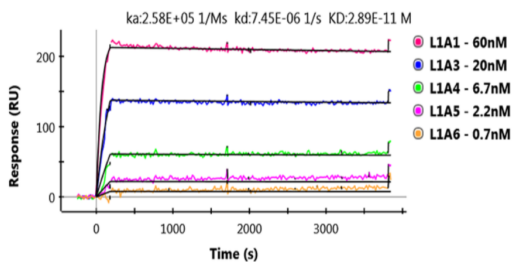

## D. 8A1

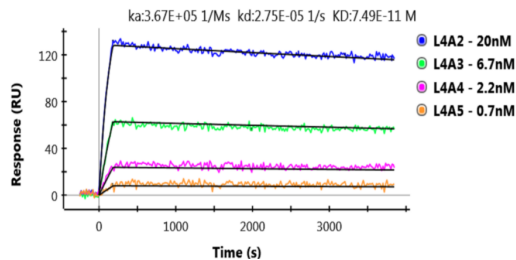

## E. LF1

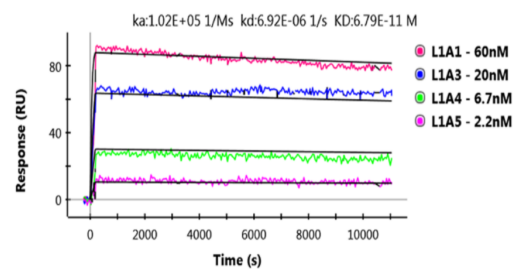

## F. 8B3

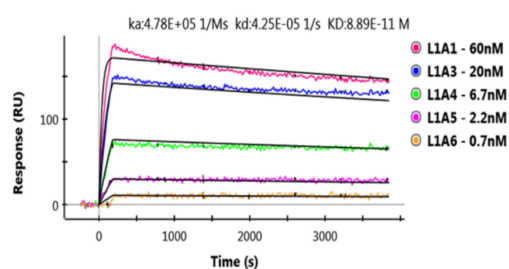

## G. LC5

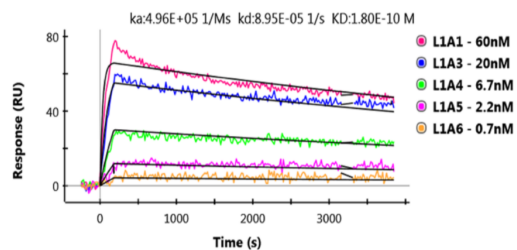

Supplement: S3 Fig — (Panels A-G Sensorgrams from SPR analysis in which ricin-coated chips (4 μg/ml) were probed with indicated RTB-mAbs. The real time binding was recorded as response units (RU) versus time. Binding was determined over a range of RTB mAb concentrations (nM); 60 (red), 20 (blue), 6.6 (green), 2.2 (purple), and 0.7 (yellow). The curves were fit using the Langmuir binding model with the ProteOn Manager software 3.1.0. (BioRad, Inc.). (PDF) [file pone.0180999.s004.pdf]

S4 Figure

A.

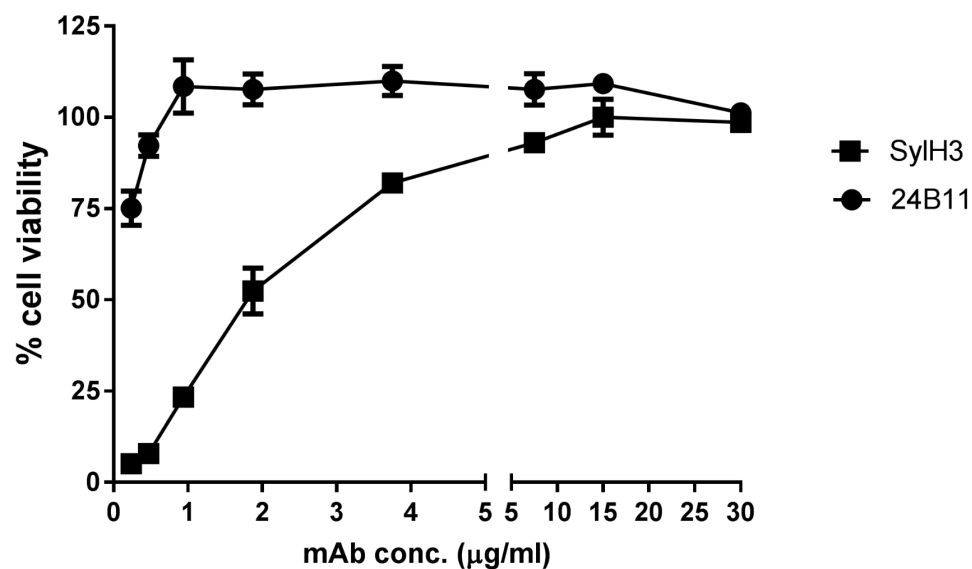

B.

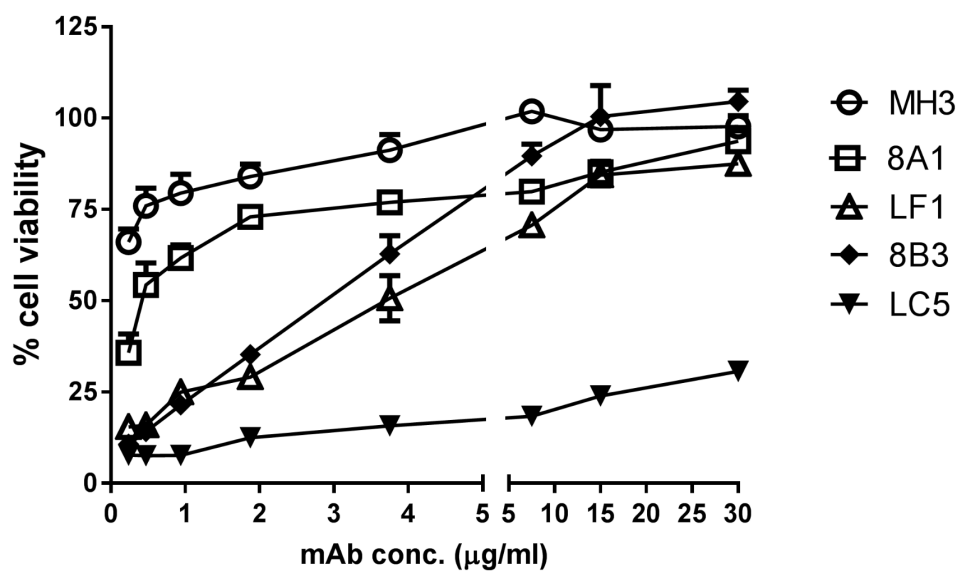

Supplement: S4 Fig — Ricin was mixed with 2-fold serial dilutions of indicated mAbs (A) SylH3 and24B11; (B) MH3, 8A1, LF1, 8B3, andLC5 and then applied to Vero cells for 2 h. The cells were then washed and cell viability was measured 48 h later, as described in Materials and Methods. The results (mean ± SD) represent a single experiment done in triplicate and repeated at least three times. As needed, the cytotoxicity assays were repeated (data not shown) to generate IC50 values. (PDF) [file pone.0180999.s005.pdf]

S5 Figure

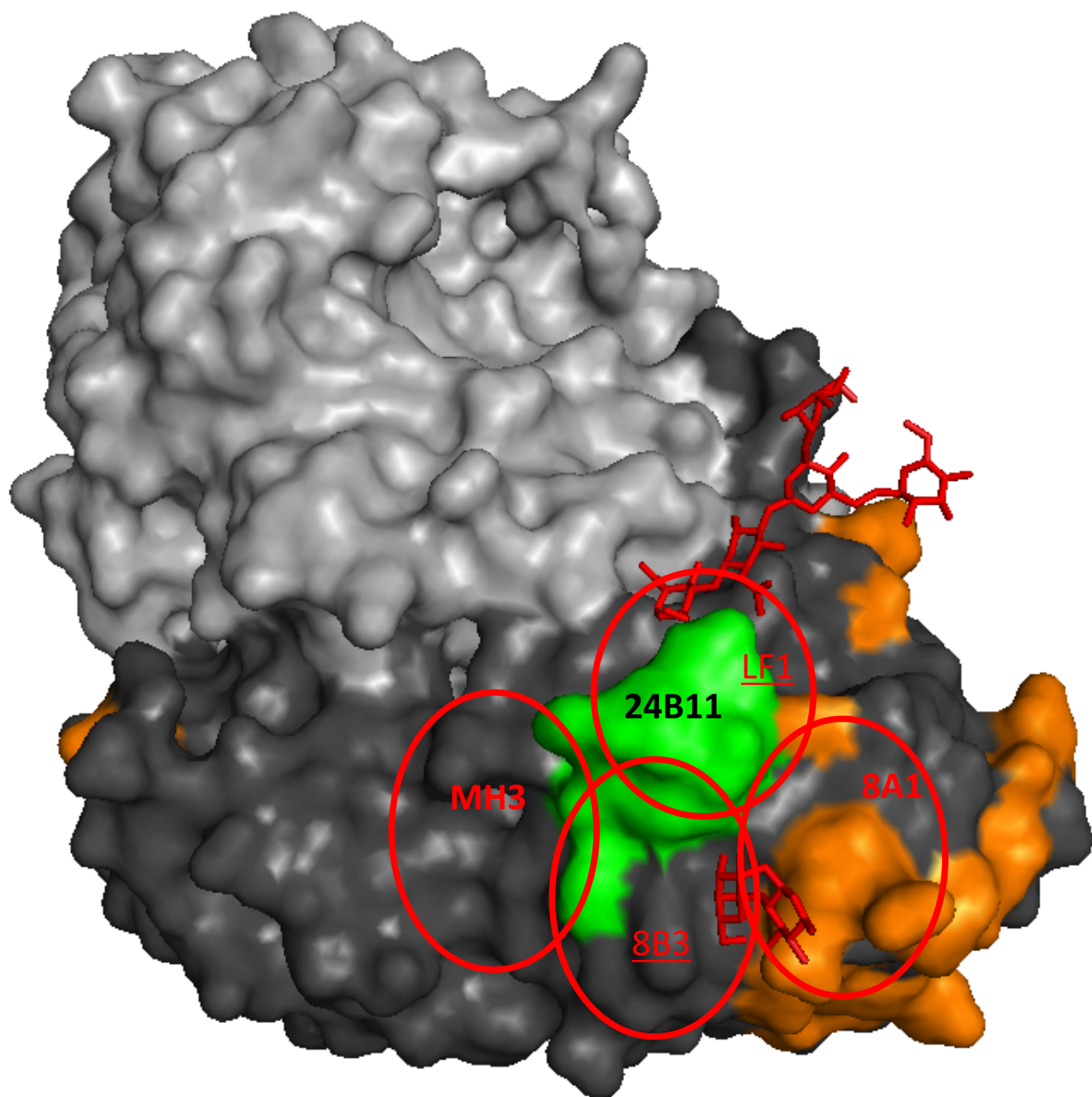

Supplement: S5 Fig — A surface representation of ricin derived using PyMol (PDB 2AAI) highlighting 24B11’s previously proposed epitope (green) and the relative spatial distribution (red circles) of epitopes recognized by MH3, 8B3, 8A1, and LF1 based on competition ELISAs presented in Table 2. Residues are non-conserved residues between RTB and RCB are colored orange. (PDF) [file pone.0180999.s006.pdf]

S6 Figure

A.

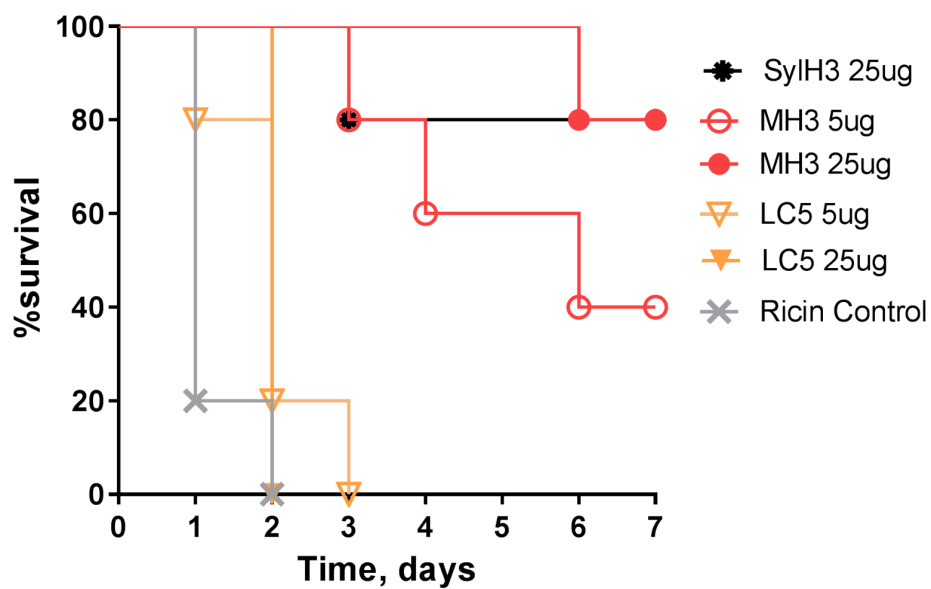

B.

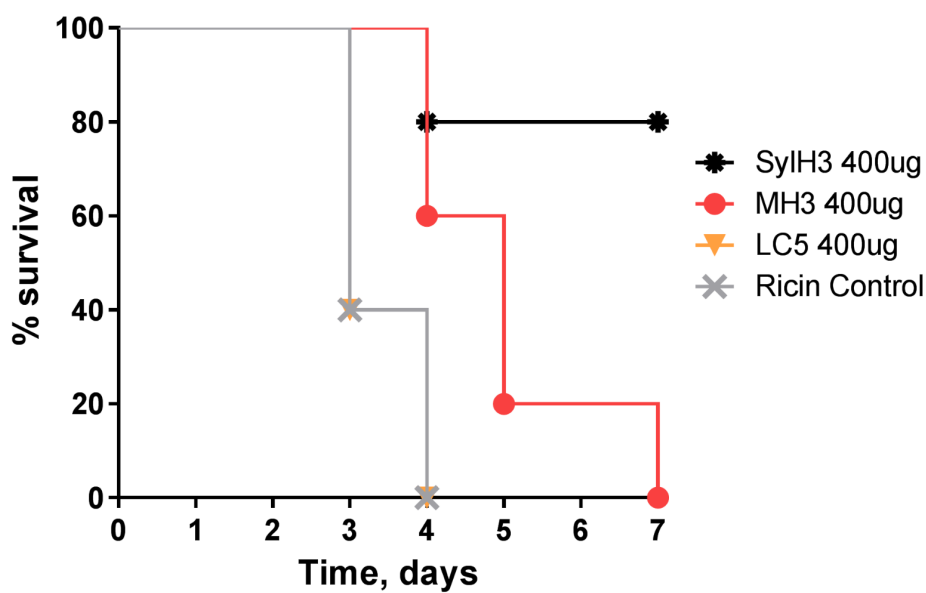

Supplement: S6 Fig — Passive protection studies in which groups of adult BALB/c mice (n = 5 per experiment) were injected intraperitoneally with indicated mAbs and then challenged 24 h later with ricin (2 μg; 10 x LD50) by the (A) intraperitoneal or (B) intranasal route. Survival was monitored for seven days. (PDF) [file pone.0180999.s007.pdf]
