# Supplementary material for: Spatial location of neutralizing and non-neutralizing B cell epitopes on domain 1 of ricin toxin’s binding subunit
Source: PLoS One. 2017 Jul 10;12(7):e0180999. doi: 10.1371/journal.pone.0180999 (PMC5507285; doi:10.1371/journal.pone.0180999)

S7 Figure

A.

	AA	*	*	*			*		*								*					*	*
**RTB-1α**	17	N	G	L	C	V	D	V	R	D	G	R	F	H	N	G	N	A	I	Q	L	W	P
**RCA1-1α**	17	N	G	L	C	V	D	V	T	G	E	E	F	F	D	G	N	P	I	Q	L	W	P
**RTB-2γ**	229	S	G	L	V	L	D	V	R	A	S	D	P	S	L	K	Q	-	I	I	L	Y	P
**RCA1-2γ**	229	N	G	L	V	L	D	V	R	R	S	D	P	S	L	K	Q	-	I	I	V	H	P

						*		*														AA
**RTB-1α**	C	K	S	N	T	D	A	N	Q	L	W	T	L	K	R	D	N	T	I	R	S	59
**RCA1-1α**	C	K	S	N	T	D	W	N	Q	L	W	T	L	R	K	D	S	T	I	R	S	59
**RTB-2γ**	L	H	G	-	-	D	P	N	Q	I	W	L	P	L	F	-	-	-	-	-	-	262
**RCA1-2γ**	F	H	G	-	-	N	L	N	Q	I	W	L	P	L	F	-	-	-	-	-	-	262

B. 1α

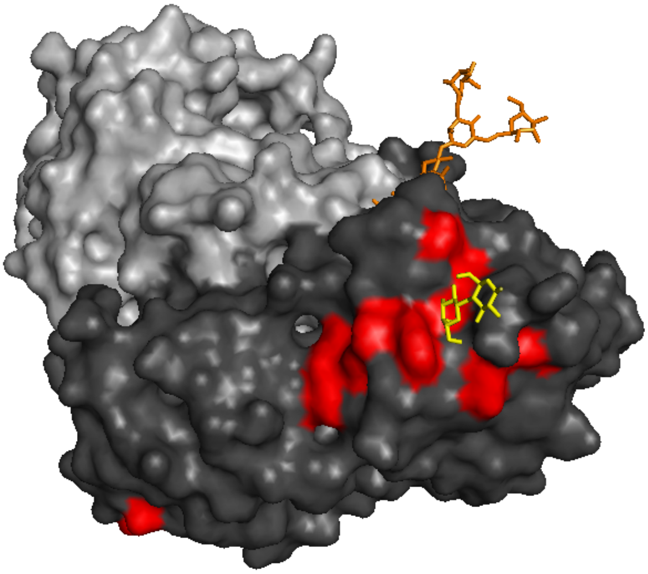

C. 2γ

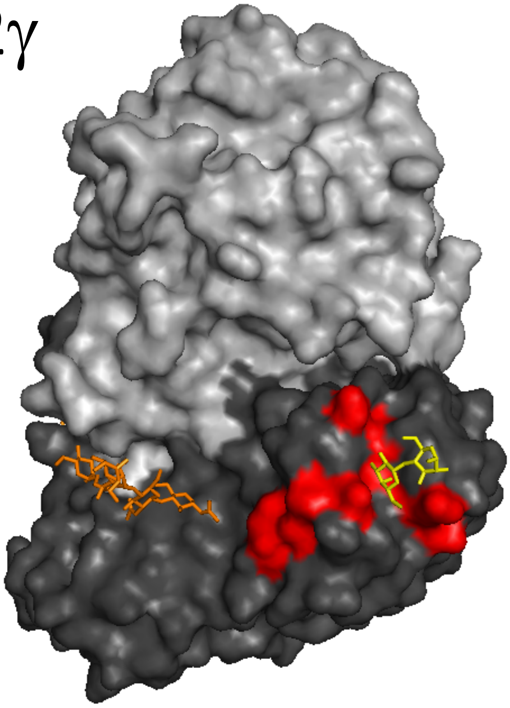

Supplement: S7 Fig — (A) Manual alignment of subdomains 1α and 2γ from RTB (PDB ID 2AAI) and RCA-I’s B subunit (RCB; PDB ID 1RZO). Boxed in bold, conserved residues; boxed, conservative substitutions; *, surface exposed residues that are conserved/conservative between RTB and RCB. These same residues are colored red in Panels B and C. (B,C) PyMol image of ricin (PDB ID 2AAI) showing potential binding sites of 8B3 on subdomains 1α and 2γ of RTB. Residues in red are surface exposed and conserved/conservative between RTB and RCB, as indicted in Panel A. (PDF) [file pone.0180999.s008.pdf]
